# Supplementary material for: Landowner perceptions of woody plants and prescribed fire in the Southern Plains, USA
Source: PLoS One. 2020 Sep 8;15(9):e0238688. doi: 10.1371/journal.pone.0238688 (PMC7478812; doi:10.1371/journal.pone.0238688)
Supplement: S1 File — (PDF) [file pone.0238688.s001.pdf]

PLEASE READ THIS PAGE FIRST

This questionnaire should be completed by the addressee or by the person who makes the management decisions for the property. **All** information you provide will remain ***strictly confidential*** and you will not be identified with your answers.

If you have any questions, please contact [redacted] by phone [redacted] or by email [redacted] or [redacted] by phone [redacted] or by email [redacted]

INITIAL QUESTION: Do you own, operate or manage 100 acres or more of rural property in Texas or Oklahoma?

- ☐ Yes → Please continue the survey beginning on the next page.
- ☐ No → If you do not own or operate a minimum of 100 acres of property in Texas or Oklahoma, please stop here and return the blank questionnaire in the postage-paid envelope provided.

It is important we hear back from everyone who receives a questionnaire, even if they do not own property. By sending the questionnaire back to us, you will be removed from the mailing list.

We thank you in advance for taking the time to complete the questionnaire and send it back to us in the enclosed, postage-paid and self-addressed envelope.

COMPLETING THE QUESTIONNAIRE:

Please make sure that you answer all questions that apply to your property.

Many of the questions in this survey use a rating scale with 7 options. Please check the box that best describes your opinion. For example, if you were asked to use such a scale to indicate the extent to which you agree or disagree with the statement that "Alaska is the best state in the USA" and you strongly agree, you would check the box furthest on the right, as follows:

	**Strongly Disagree**	**Disagree**	**Slightly Disagree**	**Neutral**	**Slightly Agree**	**Agree**	**Strongly Agree**
Texas is the best state in the USA	☐	☐	☐	☐	☐	☐	☑

If you encounter a question that does not apply to your property, please indicate this by writing "**N/A**" in the margin next to the question.

If you encounter a question for which you do not know the answer, please indicate this by writing "**DK**" in the margin next to the question.

SECTION A – ATTITUDES CONCERNING WOODY PLANTS AND FIRE

In this section we are seeking information about landowner perspectives regarding woody plants and various strategies for managing woody plants, including prescribed fire.

A1. ***To what extent do you DISAGREE OR AGREE with each of the following statements about woody plants (<u>specifically juniper/cedar and mesquite</u>)?*** FOR EACH STATEMENT PLEASE CHECK THE BOX THAT BEST REPRESENTS YOUR OPINION.

	**Strongly Disagree**	**Disagree**	**Slightly Disagree**	**Neutral**	**Slightly Agree**	**Agree**	**Strongly Agree**
Woody plants are a problem on my land.	☐	☐	☐	☐	☐	☐	☐
The increase in woody plants has reduced my ability to use my land as I would like.	☐	☐	☐	☐	☐	☐	☐
I am concerned that an increase in woody plants will increase the chance of catastrophic wildlife on my land.	☐	☐	☐	☐	☐	☐	☐
I feel the number of woody plants on my land is satisfactory.	☐	☐	☐	☐	☐	☐	☐
Cedar/juniper has increased on my property in the last ten years.	☐	☐	☐	☐	☐	☐	☐
Mesquite has increased on my property in the last ten years.	☐	☐	☐	☐	☐	☐	☐
Other woody plants have increased on my property in the last ten years.	☐	☐	☐	☐	☐	☐	☐

A2. ***If you were to look down on your land from above (e.g. aerial photo, satellite view), which picture do you feel would <u>best represent</u> the proportion of trees/shrubs (black boxes) to grassland (gray boxes) found on your property?*** PLEASE CHECK THE BOX UNDER THE DIAGRAM THAT MOST CLOSELY MATCHES YOUR PERSPECTIVE.

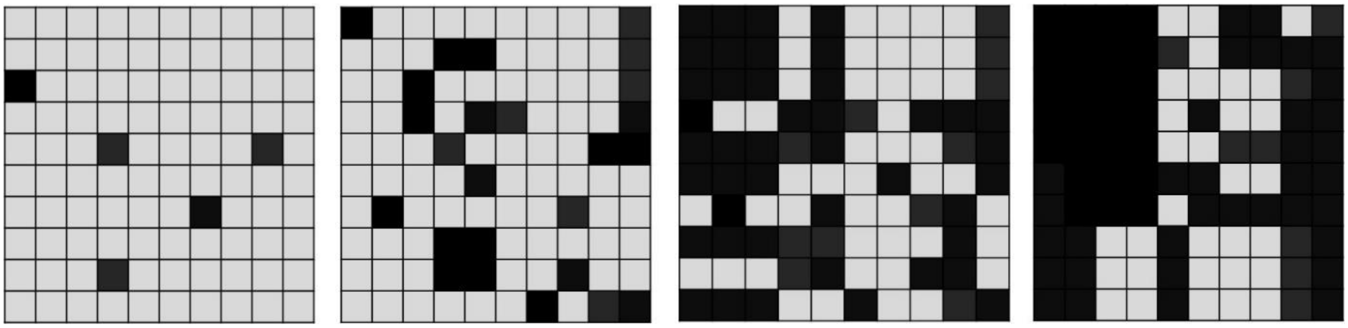

☐ approx. 5% ☐ approx. 25% ☐ approx. 50% ☐ approx. 75%+

A3. ***Using the same diagram above (A2), which proportion of trees/shrubs to grassland <u>would you prefer</u> on your property?*** PLEASE CHECK ONE BOX ONLY**.**

☐ approx. 5% ☐ approx. 25% ☐ approx. 50% ☐ approx. 75%+

SECTION C – UNDERSTANDING PRESCRIBED FIRE

In this section we are seeking detailed information on the perceptions of landowners about the use of prescribed fire as a rangeland management tool.

C1. ***Have you ever seen or heard about a prescribed fire being conducted near your property?*** ☐ Yes ☐ No

C2. ***Have you ever participated in a prescribed fire on someone else's land?*** ☐ Yes ☐ No

C3. ***Have you ever conducted a prescribed fire on your land?*** ☐ Yes ☐ No

IF YES, continue to question C3a; **IF NO**, please skip ahead to question C4

C3a. How often do you conduct a prescribed fire? *Check one.*

☐ Frequently (each year) ☐ Often (every 2-3 years) ☐ Occasionally (every 4-10 years)

☐ Rarely (>10 years between burns) ☐ I've only ever burned once

C3b. How much acreage, on average, do you burn when conducting a prescribed fire? PLEASE CHECK THE ONE BOX BELOW THAT INCLUDES THAT AVERAGE.

☐ Less than 50 acres ☐ 51-160 acres ☐ 161-320 acres ☐ More than 320 acres

C3c. Have you ever received any training on prescribed fire use? ☐ Yes ☐ No
IF YES, who conducted the training? *Check all that apply.*

☐ NRCS ☐ USFWS ☐ State Wildlife/Game Dept. ☐ Univ. Extension Service

☐ Prescribed Burn Association (PBA) ☐ Other ______________________________

C4. ***Previous litigation may affect landowners' willingness to use prescribed fire.*** PLEASE CHECK THE BOX THAT BEST FITS YOUR PERSPECTIVE ABOUT EACH QUESTION.

	Yes	No
Are you aware of any lawsuits over the use of prescribed fire in your area? **If YES, please answer the next three questions; If NO please go to question C5**	☐	☐
Has this discouraged you from using prescribed fire on your land?	☐	☐
Has this discouraged you from participating in burns on other people's property?	☐	☐
Has this affected your ability to obtain liability insurance that covers prescribed fire?	☐	☐

SECTION D – PRESCRIBED BURN ASSOCATIONS (PBAs)

In this section we are seeking specific information about prescribed burn associations in your area.

D1. ***Are you aware of any prescribed burn associations (PBAs) in your area?*** ☐ Yes ☐ No

IF YES, what is the name of the PBA?

____

D2. ***Are any of the following people you know a member of a PBA?*** *Check all that apply*.

☐ Family members ☐ Friends ☐ Neighbors ☐ Other community members

D3. ***Are you currently a member of a prescribed burn association?*** ☐ Yes ☐ No

IF YES, continue to question D4; **IF NO**, please skip ahead to Section E

D4. ***What is the name of your prescribed burn association (PBA)?*** ____________________________

D5. ***How many years have you been a member?*** ____________________________

D6. ***About how many PBA meetings do you attend per year?*** ____________________________

D7. ***In total, how many PBA affiliated burns have you participated in?*** ____________________________

D8. ***In what percentage (%) of burns you participated in was there a written burn plan?*** ________%

D9. ***In what % of burns you participated in was there a Certified Prescribed Burn Manager?*** ________%

D10. ***To what extent you DISAGREE OR AGREE with the following statements about prescribed burn associations***? IN EACH ROW PLEASE CHECK THE BOX THAT BEST REPRESENTS YOUR OPINION.

	Strongly Disagree	Disagree	Slightly Disagree	Neutral	Slightly Agree	Agree	Strongly Agree
It is important for me to be actively involved in my prescribed burn association.	☐	☐	☐	☐	☐	☐	☐
I know most of the members of my prescribed burn association.	☐	☐	☐	☐	☐	☐	☐
I consider many members of my prescribed burn association to be my friends.	☐	☐	☐	☐	☐	☐	☐
I socialize with members of my prescribed burn association.	☐	☐	☐	☐	☐	☐	☐
I trust members of my prescribed burn association.	☐	☐	☐	☐	☐	☐	☐
I would spend time helping non-family prescribed burn association members.	☐	☐	☐	☐	☐	☐	☐
I would loan equipment to non-family prescribed burn association members.	☐	☐	☐	☐	☐	☐	☐
Non-family prescribed burn association members would spend time helping me.	☐	☐	☐	☐	☐	☐	☐
Non-family prescribed burn association members would loan me equipment.	☐	☐	☐	☐	☐	☐	☐
I care what other prescribed burn association members think I should do.	☐	☐	☐	☐	☐	☐	☐
I have gotten my money's worth from participating in my prescribed burn association.	☐	☐	☐	☐	☐	☐	☐
Being a member of a prescribed burn association will help me achieve my land management objectives.	☐	☐	☐	☐	☐	☐	☐
If my prescribed burn association urged its members to adopt certain conservation practices, most would likely comply.	☐	☐	☐	☐	☐	☐	☐
If my prescribed burn association urged members to follow specific burn guidelines, I would most likely comply.	☐	☐	☐	☐	☐	☐	☐

SECTION E – LANDOWNER CHARACTERISTICS

In this section we are seeking information to classify landowners according to their motivation for owning their land and other characteristics. All of the information you provide will remain ***STRICTLY CONFIDENTIAL*** and you will not be identified with your answers.

E1. ***People own their places for many reasons. For each item below, please check the box that indicates HOW IMPORTANT each reason is to you as to why you own your property.*** IN EACH ROW PLEASE CHECK THE BOX THAT BEST REPRESENTS YOUR OPINION.

**I OWN MY PLACE TO:**	**Not at all Important**	**Unimportant**	**Somewhat Unimportant**	**Neutral**	**Somewhat Important**	**Important**	**Very Important**
Operate a farm or ranch	☐	☐	☐	☐	☐	☐	☐
Maintain family farming or ranching tradition	☐	☐	☐	☐	☐	☐	☐
Keep land in the family	☐	☐	☐	☐	☐	☐	☐
Leave land for my family	☐	☐	☐	☐	☐	☐	☐
Enjoy the outdoors	☐	☐	☐	☐	☐	☐	☐
Have a place to relax	☐	☐	☐	☐	☐	☐	☐
Be able to hunt and/or fish (recreational)	☐	☐	☐	☐	☐	☐	☐
Be able to enjoy other non-hunting/fishing recreation	☐	☐	☐	☐	☐	☐	☐
Operate a hunting enterprise	☐	☐	☐	☐	☐	☐	☐
Manage large wildlife (primarily deer)	☐	☐	☐	☐	☐	☐	☐
Manage game birds	☐	☐	☐	☐	☐	☐	☐
Produce grazing livestock (primarily cattle and/or sheep)	☐	☐	☐	☐	☐	☐	☐
Produce browsing livestock (primarily goats)	☐	☐	☐	☐	☐	☐	☐
Produce hay/forage	☐	☐	☐	☐	☐	☐	☐
Cultivate crops	☐	☐	☐	☐	☐	☐	☐
Obtain income from minerals (mainly oil and/or gas)	☐	☐	☐	☐	☐	☐	☐
Have a financial investment	☐	☐	☐	☐	☐	☐	☐
Earn a profit	☐	☐	☐	☐	☐	☐	☐
Sell the land someday at a profit	☐	☐	☐	☐	☐	☐	☐

E2. ***To what extent you DISAGREE OR AGREE with the following statements***? IN EACH ROW PLEASE CHECK THE BOX THAT BEST REPRESENTS YOUR OPINION.

	Strongly Disagree	Disagree	Slightly Disagree	Neutral	Slightly Agree	Agree	Strongly Agree
I know most of my neighbors	☐	☐	☐	☐	☐	☐	☐
I consider many of my neighbors to be friends	☐	☐	☐	☐	☐	☐	☐
I socialize with my neighbors	☐	☐	☐	☐	☐	☐	☐
In general, I trust my neighbors	☐	☐	☐	☐	☐	☐	☐
I would spend time helping neighbors conduct land management	☐	☐	☐	☐	☐	☐	☐
I would loan equipment to neighbors if needed	☐	☐	☐	☐	☐	☐	☐
My neighbors would spend time helping me with land management	☐	☐	☐	☐	☐	☐	☐
My neighbors would loan me equipment if needed	☐	☐	☐	☐	☐	☐	☐
I know a lot of people within my community	☐	☐	☐	☐	☐	☐	☐
I have a lot of friends within my community	☐	☐	☐	☐	☐	☐	☐
In general, I trust most people living in my community	☐	☐	☐	☐	☐	☐	☐
In general, people in my community help each other	☐	☐	☐	☐	☐	☐	☐
It is important for me to be actively involved in my community	☐	☐	☐	☐	☐	☐	☐

E3. ***In what year were you born?*** 19____

E4. ***What is your gender?*** ☐ Male ☐ Female

E5. ***What is the highest level of formal education you have completed?*** CHECK ONE BOX ONLY**.**

☐ Some High School ☐ High School Graduate/GED ☐ Some College

☐ Bachelor’s Degree ☐ Some Graduate School ☐ Graduate Degree

E6. ***In which county or counties is your property primarily located?*** ____________________________

E7. ***How many years have you owned your rural property as the primary owner?*** ____________

E8. ***How many years has the property been in your family (<u>including your ownership</u>)?*** ____________

E9. ***How would you describe your residency on your rural property?*** CHECK ONE BOX ONLY.

☐ Full-time resident ☐ Mainly weekend resident ☐ Occasional resident

☐ I don’t reside on my property

E10. ***Which of the following settings best describes where you grew up?*** CHECK ONE.

☐ Rural Area ☐ Small town ☐ Medium town ☐ Large town ☐ City/suburban area

E11. ***Approximately what is the acreage of your rural property?***

☐ 100-500 ☐ 501-1000 ☐ 1001-2500 ☐ more than 2,500

E12. ***In 2014, approximately what percent of your total annual income was generated from activities on your rural property?***

☐ 0% ☐ 1% to 25% ☐ 26% to 50% ☐ 51% to 75% ☐ 76% to 100%

In the space below, please share any other comments you may have about prescribed fire or woody plants on your land in general.

____

____

____

____

____

____

____

____

____

____

____

PLEASE RETURN THE COMPLETED QUESTIONNAIRE IN THE INCLUDED

SELF-ADDRESSED RETURN ENVELOPE.

THANK YOU FOR PARTICIPATING IN THIS IMPORTANT STUDY.
