# Supplementary material for: Landowner perceptions of woody plants and prescribed fire in the Southern Plains, USA
Source: PLoS One. 2020 Sep 8;15(9):e0238688. doi: 10.1371/journal.pone.0238688 (PMC7478812; doi:10.1371/journal.pone.0238688)
Supplement: Attachment — Submitted filename: Response to Reviewers.docx [file pone.0238688.s003.docx]

**PONE-D-19-20616 - Landowner perceptions of woody plants and prescribed fire in the Southern Plains, USA: *Response to Reviewer Comments***

Revision requested by 12/20/2019

- Rebuttal letter that responds to each point raised by the academic editor and reviewer(s). This letter should be uploaded as separate file and labeled 'Response to Reviewers'.
- Marked-up copy of manuscript that highlights changes made to the original version. This file should be uploaded as separate file and labeled 'Revised Manuscript with Track Changes'.
- Unmarked version of revised paper without tracked changes. This file should be uploaded as separate file and labeled 'Manuscript'.

**Journal Requirements:**

**J1.** Please ensure that your manuscript meets PLOS ONE's style requirements, including those for file naming. The PLOS ONE style templates can be found at

http://www.journals.plos.org/plosone/s/file?id=wjVg/PLOSOne_formatting_sample_main_body.pdf and http://www.journals.plos.org/plosone/s/file?id=ba62/PLOSOne_formatting_sample_title_authors_affiliations.pdf

**Response to J1**: Done

**J2.**  Please include additional information regarding the survey or questionnaire used in the study and ensure that you have provided sufficient details that others could replicate the analyses. For instance, if you developed a questionnaire as part of this study and it is not under a copyright more restrictive than CC-BY, please include a copy as Supporting Information.

**Response to J2:** We are including an abbreviated copy of the questionnaire (S1 File) which includes all of the pages that have questions we used in this particular project. We have also included the data set (S2 File) in the supporting information

**J3.** We note that you have indicated that data from this study are available upon request. PLOS only allows data to be available upon request if there are legal or ethical restrictions on sharing data publicly. For more information on unacceptable data access restrictions, please see http://journals.plos.org/plosone/s/data-availability#loc-unacceptable-data-access-restrictions. In your revised cover letter, please address the following prompts:

a) If there are ethical or legal restrictions on sharing a de-identified data set, please explain them in detail (e.g., data contain potentially sensitive information, data are owned by a third-party organization, etc.) and who has imposed them (e.g., an ethics committee). Please also provide contact information for a data access committee, ethics committee, or other institutional body to which data requests may be sent.

b) If there are no restrictions, please upload the minimal anonymized data set necessary to replicate your study findings as either Supporting Information files or to a stable, public repository and provide us with the relevant URLs, DOIs, or accession numbers. For a list of acceptable repositories, please see http://journals.plos.org/plosone/s/data-availability#loc-recommended-repositories.We will update your Data Availability statement on your behalf to reflect the information you provide.

**Response to J3:** We are including the requested minimal anonymized data set necessary to replicate our findings as a supplemental information file (S2 File)

**J4.** We note that Figures 1 in your submission contain [map/satellite] images which may be copyrighted.

**Response J4:** We have modified Figure 1 using non-copyrighted maps and added state labels to clarify per reviewer comment R.47.

**Reviewer #1:**

**R1.1: Discuss how potential bias was handled**

a. Survey recipients included 100 random landowners from each county in the study area, plus PBA members. It seems that including a specific subset of the sample population would potentially influence findings and make it difficult to draw conclusions, especially since PBA members would already understand the value of prescribed fire and therefore are potentially more aware of the presence of woody plants on their property. *How was this potential source of bias accounted for during the analyses?*

**Response R1.1a:** Using PBA membership lists, we intentionally included all PBA members in each study county because if we relied on a randomly drawn PBA membership sample drawn from the general tax records, the chance of selecting an adequately large sample of PBA members for comparison with non-member respondents would have been extremely unlikely. One of the objectives of our study was to understand the effect of PBA membership on perspectives about woody plant encroachment and the use of fire. It is unclear how PBA membership would have enhanced awareness of the presence of woody plants on their property; woody plants are equally visible to both members and non-members. It is indeed likely that PBA members have a better understanding of the use of prescribed fire; confirming this with our data is the basis for arguing that greater landowner access to PBA membership could enhance the broader use of this critically needed rangeland management tool. Previous research has also shown that many PBA members were not always supportive of the use of prescribed fire but rather that joining such a landowner organization enabled them to change their mind about prescribed fire being a risky land management tool and, consequently, to decide to apply fire on their own land (Kreuter et al. 2008). Finally, when we report and discuss our findings, we are careful to discuss only in terms of our respondents in order not to extrapolate any possible response bias to a larger population.

b. Were any other sources of bias tested for? (e.g. violations of assumptions or outliers)

**Response R1.1b:** We did not test for other potential sources of response bias. Since our data were derived primarily from specified response scales, outliers are not present. Additionally, as indicated above, to avoid extrapolating potential bias, we refer to respondents only, not landowners in general, in our presentation and discussion of results and not extrapolated to the landowner population from which the sample was drawn.

c. How were unanswered questions handled? (i.e. was there any influence created by non-responses to specific items within questionnaire areas of inquiry)

**Response R1.1c:**

We used null values for unanswered questions, resulting in some variation of n values for each variable. We based this on the assumption that data were missing completely at random (MCAR) as we could not determine respondent’s reasons for not answering any particular question. Based on this assumption, we did not impute MCAR data.

**R1.2: Manuscript organization**

a. Information in the Results section should only report results; discussion should be located in the Discussion section (line 240-243)

**Response R1.2a:** All discussion points originally included in the results section were moved to the discussion.

b. Data should be reported in the Results section (Table 7)

**Response R1.2b:** Table 7 (now Table 5) and the associated text have been moved for the results section.

**R1.3: Results relationship to Discussion and Conclusions**

a. It is important to use the Discussion and Conclusion section to show how the study complements the existing literature and answers the research questions. More of this study’s research findings should be listed, as well as referencing tables and figures from the Results sections.

**Response R1.23a:** We have expanded the discussion section to address additional significant findings (ex: age variable influence on woody cover preference and use of fire) and have connected our findings back to the literature more thoroughly, including more international literature.

**R1.4: Data/analyses transparency**

a. What software package was used for data analyses?
   **Response R1.4a:** We used STATA 13 and have included that information in the Methodology section and the works cited
b. Insert table to show questionnaire items that composed attitudes concerning woody plants and fire. This would provide context on the number and type of questions defining each construct and would be an opportunity to indicate response %. Also indicate which items were positively and negatively worded for purpose of reverse-scoring in the data analyses.

**Response R1.4b:** We have added an abbreviated questionnaire to the supplemental information so that readers can read the questions asked of participants. We only developed latent index variables for ascertaining respondent’s landownership motivations as outlined in Table 2. The question concerning woody cover preference is detailed in Table 1 under the dependent variable label and again in Figure 2 where readers can see the question prompt as it appears in the questionnaire instrument.

c. Table 2: What type of rotation was used? List rotated factor loading results for all items. Include % of Variance as a separate row.

**Response R1.4c:** Requested changes have been included in Table 2.

d. Table 5 and 6: Include standard errors for β coeff. Consider reporting odds ratio and confidence interval instead of % Δ odds, as this will help with interpretation of the results.

**Response R1.4d:** SE of β coeff. Have been included as suggested. We believe that it is simply a matter of interpretation wording preference since % change in odds is simply subtracting the odds ratio from 1 and multiplying the difference by 100. Either method (odds ratio or % change in odds) will provide the same information. Our preferred standard reporting method is % change in odds.

**R1.5: Include supporting references**

Line 284; line 296-7 (in that area – statewide?); line 315; line 241-3 (how does this relate to Table 4?); line 315-6
**Response R1.5:** Line 284 has been deleted from the manuscript per other reviewer comments. Line 296-7 reference has been added. Line 315 references have been added. Line 241-243 has been removed from the results section and moved to the discussion section and references have been added. Line 315-316 references concerning increasing wildfire risk have been added.

**R1.6: Clarity of statements**

a. line 250, 254 – be careful of interpreting % Δ odds as probability; reporting the odds ratio will allow for more accurate interpretation of data
   **Response R1.6a:** Please see response R1.4d above
b. line 282-3 – what about the possible influence of PBA member bias?
   **Response R1.6b:** Please see R1.1a above.
c. line 286 – expand on this thought
   **Response R1.6c:** We have deleted the sentence and the preceding one as these are perhaps too speculative.
d. line 303-5 – how does this species relate to the study, or include reference for why it should be specifically mentioned
   **Response R1.6d:** We have added references concerning landowner preference for retaining oak trees on their land (Olenick et al 2005) (Juzwick et al 2011)
e. line 314 – discuss how/why would it escalate wildfire risk
   **Response R1.6e:** We have added language clarifying why cedar/juniper expansion increases risk of wildfire and added 2 references as support. (Brockway et al 2009 and Twidwell et al 2013)
f. line 344 – what preceding questions?
   Response R1.6f: That sentence has been reworded for clarity

**R1.7: Completeness of reporting information**
Discuss all statistically significant findings – ex. age relationship and woody cover preference (Table 5) – all other significant findings were mentioned, including age for Table 6

**Response R1.7:** We have added language to the results section about the significant age variable in Table 5 (now Table 6)

**R1.8: Proofreading**
Several typos and grammatical errors exist and need corrected

**Response R1.8:** We have carefully proof read the revised manuscript to ensure correct grammar and eliminate typos.

**Reviewer #2:**

**R2.1:** In lines143-148 you note that you do not conduct an assessment of cover change on respondent's properties--a weakness--but you adequately attempt to qualify your results / observations.

**Response R2.1:** Since there was no recommendation for change, we did not change the text.

R2.2: L120 has an extra 'to', and presumably L129 is missing a 'not'

**Response R2.2:** Corrections included.

**Reviewer #3:**

**R3.1.** It seems odd to conduct this research on issues surrounding woody cover increase in counties that actually have had decreasing woody cover (Table 7). Was there any baseline data on woody cover and woody cover change in the study area that informed the study and choice of study sites?

**Response R3.1.** The spatial analysis of woody plant cover changes in the Southern Plains that concluded some counties experienced woody plant reduction was being conducted at the same time as the landowner survey. These assessments were two parts of a larger research project. Generally, woody plant expansion is thought to have occurred throughout the Southern Plains. We included explanations for the disparity: *“The land cover change estimates may have been influenced by the effects of a severe drought between 2010 and 2015 in that dead standing trees, which were more abundant in the Edwards Platea, may have been undetected from space whereas landowners could easily see them from ground level. Additionally, landowners are also able to see small saplings that indicate woody plant increase but these may also not be detectable from space. Conversely, some respondents may have erroneously indicated woody plant expansion because of their growing frustration with the higher than desired woody plant concentration on their land. This may be especially the case on the counties sampled in Texas which have more woody plant cover than those in Oklahoma. Additionally, Roberts et al. (2018) discovered that nearly all policies aimed at containing juniper tend to promote rather than contain the spread of the species due to “doublethink and policy-ecology mismatches.”(**17**)”*

**R3.2: Incomplete information on the questionnaire**

a. It would have been useful to have access to the whole (blank) questionnaire. This would allow readers to assess how the questions were phrased, and whether some information that would have been useful to have (e.g. reasons given for using/not using prescribed burning) was collected but not reported, or whether it was not collected. It would also allow for repeatability of the work.

**Response R3.2a.** We are including an abbreviated copy of the questionnaire in the supplemental information (S1).

b. Are there substantial aspects of the questionnaire that are not reported in this manuscript? If so, what are the reasons for this? I feel that I am getting an incomplete picture of the questionnaire and its overall aims. Are they the same as reported in this paper or is the work presented here just a subset?

**Response R3.2b.** Please see the previous response. Not all of the information from a multi- questionnaire aimed at addressing multiple research question can be presented in one journal manuscript. We provide the reference for one other publication so far above and cite it in the revised manuscript. as it includes aspects that have been published elsewhere. See: Kreuter, U.P., D.A. Stroman, C. Wonkka, J. Weir, A.A. Abney, J.K. Hoffman. 2019. Landowner perceptions of legal liability for using prescribed fire in the Southern Plains, USA. Rangeland Ecology & Management. 72: 959-967. This very recent publication has been added in the discussion of liability concerns.

c. It would have been useful to have asked farmers to estimate the woody cover on their farms at some earlier date using the sample schematics used to elicit perceptions of present and desirable woody cover.

**Response R3.2b.** We did ask survey participants to respond to the following three statements, although they were not asked to refer to the schematic in selecting their response option, which could have been beneficial: Cedar/juniper has increased on my property in the last ten years; Mesquite has increased on my property in the last ten years; Other woody plants have increased on my property in the last ten years. We report this information in the revised manuscript.

d. The authors state that the survey data cannot be freely shared because of ethics/confidentiality concerns, but this restriction should not apply to the actual questionnaire that was used.

**Response R3.2d.** Please see Response 3.2a. and 3.2b.

**R3.3: Potential sampling bias resulting from the study design**

a. The authors simply state that “A non-response bias analysis was not conducted because alternative contact information (e.g., telephone number) required for such an analysis was not obtainable. Due to this limitation, we do extrapolate our results to all landowners in the Southern Plains.” (lines 127-129). This sounds a bit like "we couldn't do it, therefore it was not necessary", but the possible response biases (e.g. age) and their likely an influence on the findings needs to be properly acknowledged and dealt with. The statistical analyses used assume that the data represent a random sample from the population, and if this assumption is violated then the inferences drawn from the sample cannot be applied to the population.

**Response R3.3a.** See response in R3.3c. We also changed the wording in line 127-129 to read “Due to this limitation, we do **not** extrapolate our results to all landowners in the Southern Plains.”

b. I identified two (possible) sources of sampling bias that are likely to have influenced the results of the analyses and these need to be better discussed and taken into account when drawing conclusions from the research.

**Response R3.3b.** See response in R3.3c

c. The first is that the overall sample was chosen as a random subset of 100 landowners in a county, but to this the authors added 318 purposively chosen members of PBAs (making up 16.6 % of the total questionnaires mailed and > 30 % of the responses received). The authors do not tell the reader what percentage the 100 randomly selected landowners per county represents of the total eligible population (landowners with a minimum 100 acre property size). Similarly, it is not clear what percentage of PBA members is represented by the 318 PBA members that were contacted. Including the PBA members as part of the otherwise random sample violates the assumptions of the regression analyses that used to analyze the influence of various demographic factors on woody cover preference and use of fire. The magnitude of the possible bias this introduces is impossible to estimate since no information is given on the overall ratio of PBA members to the general population, and how this compares to the ratio of respondents in the data (> 30 % of the survey respondents were PBA members who were purposively selected as survey participants).

**Response R3.3c.**

First, it is impossible to report the total eligible population of landowners in a county who own 100 acres of land or more because many landowners own multiple plats that equal or are greater than 100 acres. We compiled a list of all landowners, according to county tax records, who met the minimum acreage threshold, eliminated any duplicate names and pulled our sample from there. Second, had we relied only on the county tax records to randomly draw landowners who are PBA members, we would have obtained a very small sample of such landowners. Since we were interested in exploring differences in prescribed fire perspectives between PBA member and non-member landowners, we needed to ensure an adequate number of PBA member landowners in our study. Therefore, whereas the non-member landowners represent a random sample of landowners who own at least one plat of land = > 100 acres, the survey participants who were PBA members represent the entire population of PBA members who reside in the counties we surveyed.

d. Was there any exploration of what led PBA members to become members of PBAs? One of the study’s findings if that PBA members were more likely to have used fire in the past, but what can one infer about cause and effect? Would landowners given to using fire not be more likely to join a PBA in the first place? Or does joining a PBA result in landowners being more comfortable using fire?

**Response R3.3d.** In this study, we did not inquire about motivations for becoming a PBA member. Previous research indicates that membership motivations vary including safe fire training, access to fire management equipment on burn days, assistance with application of prescribed fire, and access to prescribed fire liability insurance. However, we did ask some questions about how the PBA influences their social networks. While we do not know from this study if joining a PBA resulted in more landowners being more comfortable using fire, previous research (Kreuter et al. 2008, Toledo et al. 2014) as well as this study show there is strong correlation between PBA membership and application of fire on the members own property and assistance with the application of prescribed fire on other people’s properties.

e. The second possible source of bias is the use of a mail survey. I don’t know what the current situation is in the rural areas of Texas and Oklahoma, but in many parts of the world few people read and respond to letters via “snail” mail. There are likely to be valid reasons for choosing this method, but they need to be justified and explained, and possible biases that may have arisen need to be discussed, ideally with reference to the international literature. I would expect, for example, that older landowners would be more likely to respond to a mail survey. The sample is very strongly skewed towards older respondents but it is unclear whether this reflects the demographics of landowners in the area or whether this is a respondent bias part of the survey. If the latter then the survey results may under-represent the perceptions, preferences and practices of younger farmers who are significantly different in these respects. PBA members made up a third of respondents, what is their age profile compared to that of the non-PBA members? At the very least, the authors should make an effort to compare this to the actual demographics of the study area, which should be available from population census or similar data, to see if a significant age bias exists.

**Response R3.3e.** We have added information about age to the discussion, including several references. The reported average age (66) is in line with several similar studies both within our study area and throughout the U.S. (see references in discussion section). Our age range of respondents was between 30-93, which indicates that the use of a mail survey is still an accessible method for reaching private landowners of all ages. County land ownership databases in our study area do not include information such as phone or email contacts that may allow for other survey methods. In addition, older landowners may be less likely to answer long form surveys by phone or email. Moreover, we have conducted mail surveys for over 20 years with consistently satisfactory response rates ranging from 30-50% whereas the email/web-based surveys that we have conducted have resulted in extremely low response rates of 5% or less.

f. Both these sources of (possible) bias have a bearing on the results reported. Age was a significant predictor of woody cover preference, and both age and PBA membership were found to significantly predict use of prescribed burning. Without consideration (and where possible, elimination) of sampling bias, these results cannot be considered to be robust.

**Response R3.3f.** Please see R1.1a above.

**R3.4: Inclusion of woody cover trend data from Hartfield & van Leeuwen (2018)**

a. The obvious gap when trying to determine whether farmer’s perception of current woody cover and past change are accurate is some objective measure of actual cover. The authors address this by using data from a remote sensing study (Hartfield & van Leeuwen 2018) to compare perceptions of change to published rates of change on a per-county basis. While this is a good idea, there are several problems with how this was done and reported.

**Response R3.4a.** See response R3.4c

b. The first issue concerns the scale mismatch between the perceptions at the individual landowner level and the change data at the level of the county. The authors explain that this was unavoidable, but the implications are not adequately explored. No data of actual woody cover in each county, and some indication of how variable it is in space, are presented. If woody cover in a county varies a lot spatially, then linking it to individual landowner responses will be a bit of a stretch.

**Response R3.4b.** See response R3.4c

c. The survey was done in 2015, but the study that is cited as the source of the actual change data was published a couple of years later in 2018. I assume that the remote sensing study therefore did not inform the choice of counties for conducting the survey, but that the data were later accessed to compare perceptions of change to actual change. This is a pity, since the motivation for the survey was that woody encroachment is a significant issue, whereas the remote sensing study found that many counties did not actually experience an increase in woody cover. Possible reasons for the discrepancy between landowners’ perceptions are discussed, but in a rather speculative fashion.

**Response R3.4c.** We did not have access to the woody cover data cited in the paper during project design. However we have added data from the same source as the % cover change about actual woody plant coverage by county into Table 5.

d. I was unable to ascertain where the authors obtained the data shown in Table 7. No source is given in the table caption, but the source is indicated in the text to be Hartfield & van Leeuwen (2018). I looked at the Hartfield & van Leeuwen (2018) paper and the data do not appear in it. Instead, Hartfield and van Leeuwen state in their discussion that “In a future paper, we will use estimates of woody cover from two dates, to compute the change in woody cover for each pixel in the two ecoregions.” So where do the data cited in the submitted manuscript come from?

**Response R3.4e**. The data were obtained directly from Hartfield and van Leeuwen, who were Co-PIs on a larger project of which our survey was one of our components.

e. Since the authors did manage to access woody change cover for the counties in which their survey was done (and will hopefully be able to provide a correct citation), they should also have been able to source and report woody cover in 2004 and 2014, the latter being the year before the survey. This would allow not only comparison of woody cover estimates and actual woody cover, but also whether actual woody cover in a county influenced perceptions and use of prescribed burning.

**Response R3.4e.** We have added in the woody cover estimates for 2002 and 2014 from the same source as the % change in woody cover. However, we elected not to add county cover estimates into our models concerning perception and use of fire because some respondents may own property in other counties and accordingly their perceptions are not informed only from woody plant cover in the counties in which their sampled property is located.

f. Lastly, the perceptions of trends and the comparison with published woody cover trends should have been reported in the results section. Instead, they come as something of a surprise in the form of a table and accompanying text at the beginning of the discussion section. The discussion should not introduce new findings but needs to interpret the findings reported in the results section.

**Response R3.4a.** These results have been moved to the results subsection, “Woody plant cover perceptions and preferences.”

**R3.5:** For publication in an international journal, the introduction is rather narrowly focused on a local problem. I would have liked to see more international literature to contextualize the situation in Great Plains. The discussion should similarly draw conclusions that go beyond the regional.

**Response R3.5.** We have added more international literature to our discussion in order to draw comparisons with similar issues globally, e.g., Sutherland et al. 2011; Eburn and Cary 2018.

**R3.6:** Line 32: Were landowners just asked about the “amount” or also about perceived trends?
**Response R3.6.** They were also asked about their perceptions about increases in woody plant cover on their land. See R3.24 for more details.

**R3.7:** Lines 36 and 82: “lability” should be “liability”
**Response R3.7.** Done

**R3.8:** Line 39: Does membership of PBA cause landowners to be more likely to use fire, or do landowners who are comfortable using fire join PBAs more readily?
**Response R3.8.** That is a good question. Because of the requirement by many PBAs for members to assist with burns on other members properties before they receive assistance with a burn on their own property, the first alterative is more likely, i.e., that PBA cause landowners to be more likely to use fire. This is emphasized in the discussion where the following sentence has been added: “it has been found that that PBA’s are very effective in engaging landowners in the use of prescribed fire, because they provide their members with fire safety training and equipment and they promote safe fire application experience among members who assist each other on burn days.” We also added another reference to support this point.

**R3.9:** Lines 44-46: This final sentence of the abstract is rather vague.
**Response R3.9. The sentence has been changed to: “**Future outreach efforts to promote prescribed fire to maintain grasslands should more actively support prescribed burn associations, which an effective vehicle for increasing prescribed fire use by private landowners.”

**R3.10:** Line 50: The values of woody cover increase given here contrast with the declines in woody cover reported in Table 7.
**Response R3.10.** To address this issue, we come back to the trends reported by Berg et al. 2015 (Ref #1) in discussing the information derived from Hartfield & van Leeuwen (2018) and presented in Table 5.

**R3.11:** Lines 55-56: Use the singular (“climate change” and “land use”)?
**Response R3.11.** Done

**R3.12:** Lines 59-61: Why were PBA members explicitly included in the survey while membership of FMAs and the other organizations cited was seemingly not considered or explored at all? Also, since FMAs and NRCS are never mentioned again, there is no need to introduce the abbreviations here.
**Response R3.12.** To avoid confusion, we eliminated the term Fire Management Associations (FMAs), because it is equivalent to Prescribed Burn Associations (PBAs), which is the more generally used term and the term we use throughout the manuscript. We also deleted (NRCS).

**R3.13:** Line 71: The argument about under- or overestimating woody cover is valid but in the absence of actual woody cover data, the study does not actually determine whether farmers under- or over-estimated woody cove on their land.
**Response R3.13.** We changed the sentence to: “Accurately estimating woody cover density is difficult from ground level because visual foreshortening of dispersed trees seen from that perspective may suggest higher woody plant density than overhead measurements of canopy cover would indicate.”

**R3.14:** Lines 84-85: The first question is about landowner perceptions about woody plant expansion and how it correlates with actual trends but much of the focus of the study (and the reported analyses) is farmer’s estimation of current woody cover (rather than trend). “Trend” and “current cover” are not always clearly distinguished in the writing.
**Response R3.14.** We changed “actual trends” to “estimated changes” which more accurately represents the data we presented in Table 7. We did not use the term “current cover.

**R3.15:** Lines 96 and 99: Change “included” to “were”. “Included” implies that the subsequent list is a subset, when in fact all counties sampled are listed.
**Response R3.15.** Done

**R3.16:** Lines 116-117: What were the “five areas of enquiry including attitudes concerning woody plants and fire” and are they all reported on in this manuscript? If not, why not?
**Response R3.16.** Given that the not all 5 areas of inquiry are presented in the manuscript, we deleted this sentence as it creates ambiguity.

**R3.17:** Line 119 and elsewhere: Journal requirements include the use of SI units. Acres are not SI units. The values given need to be converted to hectares, or at least report equivalent values in SI units for the benefit of readers accustomed to the metric system.
**Response R3.17.** All property sizes were changed to ha with the acres shown in parentheses.

**R3.18:** Line 119: What proportion of landowners with properties > 100 acres does the sample of 1600 represent?
**Response** R3.18. 100%. Landowners with less than 100 acres were disqualified from inclusion.

**R3.19:** Line 120: What is the basis for the 100-acre minimum? Can the choice of this minimum value, and the assumption that owners of smaller properties would be less likely to use fire, somehow be substantiated (e.g. with a reference from the literature)?
**Response R3.19. As we stated in the manuscript: “**We applied the minimum acreage requirement with the assumption that landowners with smaller acreage properties are less likely to apply prescribed fire …”. This was based on information gathered during pre-survey meetings with personnel from the Natural Resources Conservation Services, Texas A&M AgriLife Extension, an Oklahoma Extension Service.

**R3.20:** Line 123: “Pulled from” seems rather colloquial. “Extracted from”?
**Response R3.20.** Done.

**R3.21**: Lines 124-125 – what percentage of PBA members do the 126 / 192 members from TX /OK represent?
**Response R3.21.** All identified PBA members in each study county were included in the study. If we had relied only on the sample extracted from the county tax records, we would have had a very small number of PBA-member respondents, which would have prevented us from making member/non-member comparisons.

**R3.22**: Line 131: “Participants” needs an apostrophe.
**Response R3.22.** Done

**R3.23**: Line 132: Close bracket after “trees”.
**Response R3.23.** Done

**R3.24**: Line 143: Was this a “yes/no” type of question?
**Response R3.24.** We used the 7-point response scale to answer the question: “To what extent do you DISAGREE OR AGREE with each of the following statements about woody plants?” … with respect to increases in juniper, mesquite and other woody plants during the last 10 years. This is now evident in the survey questionnaire we have added in the Supplemental information.

**R3.25**: Line 178: Should “assumption” be replaced with “prediction” or “hypothesis”?
**Response R3.25.** We replaced “assumption” with “prediction”.

**R3.26**: Table 3, last row: OK is abbreviated here but this is the only instance – keep use of abbreviations consistent or avoid them.
**Response R3.26.** Oklahoma has been spelled out throughout the manuscript.

**R3.27**: Lines 198-199 – how do the sample demographics compare to the general population? Also, all results should be consistently written in the past tense.
**Response R3.27.** We are uncertain how we should report general population statistics. We could report general demographic statistics for the 16 study counties or for each of the two states but do not see what this would add to the manuscript. Suffice it to say, that the demographic results provided approximate those from a plethora of other surveys conducted by the researchers during the last 20 years. The past tense has been used throughout in the landowner characteristics results.

**R3.28**: Line 204: But this figure is influenced by the fact that PBA members were actively targeted. What is the rate of PBA membership actually like among landowners?
**Response R3.28.** We could not determine which figure in line 204 the reviewer is referring to. No figure was referenced in that line or on that page or the subsequent page.

**R3.29**: Table 4. It would be useful to also include what percentage of respondents fall within the "ranching", "heritage", "hunting" and "farmer" land use categories.
**Response R3.29.** They are not landowner categories they are motivations that landowners have for owning their land. Each respondent may fit into more than one motivation category. We have changed the manuscript to reflect that they are landownership motivations and not categories of landowners (see table 6 and 7). The mean response score for each motivation is also presented in Table 2 to give readers an idea of which motivations were more common. For example, lifestyle/recreation motivations were overall high compared to the farming motivations.

**R3.30**: Paragraph following Line 207: It would be useful to include the published data on woody cover and trends in each county here and analyze the differences by county.
**Response R3.30.** Table 7 (now Table 5) and the associated text have been moved to this subsection to address this comment R3.39.

**R3.31**: Line 235: Did the questionnaire not probe landowners’ reasons for using / not using fire?
**Response R3.31.** We did not see how this comment relates to the statement on line 235.

**R3.32**: Line 235: What is meant by “legitimate” in this context?
**Response R3.32.** The line reference provided should have been 325. We changed the “legitimate” to “safe and effective.”

**R3.33**: Line 240: "Reported" is ambiguous – does this refer to woody cover reported by respondents or in the literature? Would “perceived” not be a more appropriate word here than “reported”?
**Response R3.33.** Reported was changed to perceived.

**R3.34**: Tables 5 and 6: p-values are not 0.000 – they are greater than zero and should be presented as < 0.001.
**Response R3.34.** Done

**R3.35**: Line 248: “low-cost, effective”: Is that how it was put in the questionnaire? If not, I would leave these value-laden terms out of the results section.
**Response R3.35.** Survey participants were asked the extent to which they agreed with the notion that prescribed fire is “more effective” and “less costly” “than other woody methods for controlling woody plants.” This language was based on the finding by Van Liew et al. 2012, which is cited and refenced in the manuscript. We have added “relatively” to the sentence to connect it to that paper, which is now also cited in this sentence to support the statement.

**R3.36**: Line 250: delete “By contrast”.
**Response R3.36.** Done

**R3.37**: Lines 255-256: Rather state this more intuitively, e.g. "older land owners were significantly less likely to have used fire...". The sentence needs a full stop.
**Response R3.37.** Done.

**R3.38**: Lines 261 onwards: This needs to be reported in the results section, not the discussion.
**Response R3.38.** Done

**R3.39**: Table 7 needs to cite the source of the woody cover change data. Also include actual woody cover in 2004 and 2014 for each county, as these values are of interest. For example, a small decline in woody cover might be outweighed in people’s perceptions by overall high woody cover values.
**Response R3.39.** The source of the woody cover change date was already cited as reference #14 in footnote b. The 2004 and 2014 woody plant cover data have also been added to Table 7 (now Table 5).

**R3.40**: Line 279: Any data / evidence / literature to support this?
**Response R3.40.** We have added three references about the effect of drought on woody plant mortality. They are Twidwell et al. 2014, Moore et al. 2016 and Croucher et al. 2019.

**R3.41**: Line 283: farmers may be frustrated by the woody cover, but the reader is frustrated by the absence of data on actual woody cover.
**Response R3.41.** We have deleted the statement. We did provide data about county level woody plant cover (Table 5) as well as landowner perceptions of woody cover on their own land, based on Figure 2. Measuring actual woody cover on each respondent’s land would be extremely difficult and beyond the scope of this study. For this reason, we obtained information about landowner perception of woody plant cover on their land.

**R3.42**: Lines 285-287: What does that mean? Not very enlightening when put like this unless one goes and reads the paper (beyond its title). Not only is it unclear what argument the cited paper makes, but there is also no explanation of how/why this would influence perceptions of actual woody cover.
**Response R3.42.** We have deleted the statement as it relates to the previous sentence.

**R3.43**: Lines 295 and 297: It would be useful here to know what the percentage of landowners of the rancher, farmer, heritage, hunter types was in in each state.
**Response R3.43.** These percentages have been included in Table 4.

**R3.44**: Line 298: Replace the question mark with a full stop.
**Response R3.44.** Done.

**R3.45**: Line 322: From what I can ascertain, actual willingness was not measured – “used fire more frequently” would seem more accurate here than “more willing”.
**Response R3.45.** We changed “more willing” to “appeared to be more accepting” as we did not measure frequency of prescribed fire use either.

**R3.46**: Line 332: How about training and support in the use of fire?
**Response R3.46.** The sentence has been modified to included this suggestion.

**R3.47**: Figure 1: For the benefit of an international audience, please label the states of Texas and Oklahoma.

**Response R3.47.** We have modified Figure 1, including labeling Texas and Oklahoma.
