# Supplementary material for: Landowner perceptions of woody plants and prescribed fire in the Southern Plains, USA
Source: PLoS One. 2020 Sep 8;15(9):e0238688. doi: 10.1371/journal.pone.0238688 (PMC7478812; doi:10.1371/journal.pone.0238688)
Supplement: Attachment — Submitted filename: PONE-D-19-20616R1 Reviewer Response.docx [file pone.0238688.s004.docx]

**PONE-D-19-20616R1 – Response to Reviewer Comments**

**Comment AE 1.1:** The issue of non-random participation in the PBA was raised during the first review and it has not been addressed in this revised version.  I agree with this concern clearly emphasized by Reviewer 3. One statistical tool to compensate for the non-random nature of participation is the use of propensity score matching. Within STATA 13, which the authors are using, this is relatively simple to do these days. Please see:[https://www.stata.com/manuals13/teteffectspsmatch.pdf](https://urldefense.proofpoint.com/v2/url?u=https-3A__www.stata.com_manuals13_teteffectspsmatch.pdf&d=DwMF-g&c=u6LDEWzohnDQ01ySGnxMzg&r=zG3MPpyaRdJlG6uGAIm77Q&m=vb4NfJlJUMnA_KoOth5QOyBrU-NtDMLlmZ82izz8cxU&s=xpcF2Rn185EIbjIzocFoHh7IiFjgM-8DuOqaQ2Pm0AA&e=). This step will strengthen the study tremendously. The author should, nonetheless, see this as a suggestion and may take a different analytical approach when addressing the issue of non-random participation.

***Response AE 1.1:*** *To address the ongoing concern about non-random participation of PBA members, we have rewritten the description of our sampling approach as follows:* ***“****The survey included 1,918 landowners in Texas and Oklahoma who owned a minimum of 40.5 ha (100 acres). We applied this minimum size requirement with the assumption that landowners with smaller sized properties are less likely to apply prescribed fire, regardless of their perspectives of woody plant density. This survey sample was derived using a stratified sample selection approach. The first stratum consisted of general landowners included in the tax databases of each of the 16 selected counties. From each of these databases, 100 landowners who owned at least 40.5 ha were randomly selected for a first stratum sub-sample of 1,600 landowners. The second stratum consisted all members of PBA in the 16 selected counties. The population of PBA members in each of the two states was small enough that our PBA sample consisted the whole subgroup. This resulted second stratum sub-sample of 318 PBA members (126 in Texas and 192 in Oklahoma). To ensure that the PBA members were not a biased subset of the combined sample of 1,918 landowners, we compared responses from the two subsamples regarding perceptions of woody plants and prescribed fire and about the proportion of income derived from the property.”*

*The results of this analysis are included at the beginning of the results section. We found no significant difference between PBA members and non-members regarding the proportion of annual income derived from the property or about existing and preferred woody plant density (see Table 4 in the manuscript). However, PBA members were more likely to respond that woody plants are a problem on their land (non -members also agreed with that statement but not as strongly), and they were also much more likely to respond that they were in favor of applying prescribed fire to their own land. In the discussion we included the following statement to address these results: “While we did not find any difference between PBA members and non-members in their perceptions and preferences of woody plant densities on their land, we did find that PBA members were more likely to view woody plant expansion as a problem on their property and to apply prescribed fire to address this concern. However, the directionality of this difference is unclear; i.e., whether PBA membership elevated the perception of woody plants as a problem or if landowners who viewed woody plants as a problem were more likely to become a PBA member in order to apply fire using PBA membership benefits including access to labor, fire management equipment and fire training.”*

**Comment AE 1.2:** The authors seem to dismiss the comment raised by the reviewer regarding non-response bias by indicating that because non follow-up phone calls were made this cannot be tested. The authors could conduct a simple comparison between early respondents and later respondents (e.g. first and second waves) and infer non-response bias from differences between these two groups. See: Armstrong, J.S., Overton, T.S., 1977. Estimating non-response bias in mail surveys. Journal of Marketing Research 14 (3), 396–402.

***Response AE 1.2:*** *As previously stated, we were not able to follow up with non-respondents so conducting traditional non-response tests was not possible for this study. Per the reviewers’ recommendation based on Armstrong and Overton (1977), which has been cited over 15,000, we have now attempted to test for potential non-response bias by comparing responses from early and late respondents, even though we could find no evidence that this method has been quantitatively verified to support it as an accurate estimate of non-response bias. Respondents were included in one of two groups based on receipt of their questionnaire during or after the first 3 weeks after the mailing date of the questionnaire (i.e., first and second wave responders). We used Mann-Whitney rank tests to compared the variable values of the two groups for five key variables: age of respondent; reliance on income from their property; perception of and preference for woody plant cover on their land, and whether or not they thought woody plants were a problem on their land. We found no statistically significant differences for any of the five variables between the two groups (p = 0.0707 - 0.7721). However, we are not convinced that testing early versus later respondents is a legitimate method for dealing with non-response bias as all the data are from respondents. Therefore, we have continued to be cautious about extrapolating our results to the broader population of landowners in the 16 counties from which the sample of general landowners was drawn. We have included this concern in the limitations paragraph in the discussion section.*

**Comment AE 1.3:** The authors shall address the minor issues noted by reviewer 1.

***Response AE 1.2: These responses are provided below.***

**Comment R 1.1:**  It still appears there is potential sampling bias. When a specific subset of a larger population (PBA members) is sampled and responses combined with a random sampling of the larger population (randomly selected landowners), there is the possibility that the responses from the subset will influence the data set when all responses are analyzed together. It would be more appropriate to analyze each population separately and compare responses from each demographic. If one dataset is used for analyses then a clear description of how this would not influence the results needs to be stated.

***Response R 1.1:*** *See response AE 1.1. above.*

Previous Comment R1.1: Discuss how potential bias was handled
a. Survey recipients included 100 random landowners from each county in the study area, plus PBA members. It seems that including a specific subset of the sample population would potentially influence findings and make it difficult to draw conclusions, especially since PBA members would already understand the value of prescribed fire and therefore are potentially more aware of the presence of woody plants on their property. How was this potential source of bias accounted for during the analyses?
Previous Response R1.1a: Using PBA membership lists, we intentionally included all PBA members in each study county because if we relied on a randomly drawn PBA membership sample drawn from the general tax records, the chance of selecting an adequately large sample of PBA members for comparison with non-member respondents would have been extremely unlikely. One of the objectives of our study was to understand the effect of PBA membership on perspectives about woody plant encroachment and the use of fire. It is unclear how PBA membership would have enhanced awareness of the presence of woody plants on their property; woody plants are equally visible to both members and non-members. It is indeed likely that PBA members have a better understanding of the use of prescribed fire; confirming this with our data is the basis for arguing that greater landowner access to PBA membership could enhance the broader use of this critically needed rangeland management tool. Previous research has also shown that many PBA members were not always supportive of the use of prescribed fire but rather that joining such a landowner organization enabled them to change their mind about prescribed fire being a risky land management tool and, consequently, to decide to apply fire on their own land (Kreuter et al. 2008). Finally, when we report and discuss our findings, we are careful to discuss only in terms of our respondents in order not to extrapolate any possible response bias to a larger population.

**Comment R 1.2:**  Providing information on potential bias and how it was handled will help to ensure that results from the data analyses are valid and accurate. Not only does this include outliers (within the data set, not just outside of the specified response scale), but violations of assumptions of statistical tests (e.g. additivity and linearity; normality; homoscedasticity/ homogeneity of variance; independence). This information might also help address potential sampling bias.

***Response R 1.2:*** *See response AE 1.1 and AE 1.2 above. We do not understand what the reviewer means by outliers within the data set – we had previously stated that the response data were primarily based on specified response options (generally 5).*

Previous Comment R1.1b: Were any other sources of bias tested for? (e.g. violations of assumptions or outliers)
Previous Response R1.1b: We did not test for other potential sources of response bias. Since our
data were derived primarily from specified response scales, outliers are not present.
Additionally, as indicated above, to avoid extrapolating potential bias, we refer to
respondents only, not landowners in general, in our presentation and discussion of results
and not extrapolated to the broader landowner population from which the sample was drawn.

**Comment R 1.3:**  Again, this is information that should be included in the manuscript.

***Response R 1.3:*** *We have included this information in the methods section of the manuscript as suggested.*

Previous comment R 1.1c. How were unanswered questions handled? (i.e. was there any influence created by nonresponses to specific items within questionnaire areas of inquiry)
Previous Response R1.1c: We used null values for unanswered questions, resulting in some variation of n values for each variable. We based this on the assumption that data were missing completely at random (MCAR) as we could not determine respondent’s reasons for not answering any particular question. Based on this assumption, we did not impute MCAR data.

**Comment R 1.4:**  Grammatical errors and typos persist; continue to revise. Replace colloquial wording with specific language.

***Response R 1.4:*** *We have again proofread the entire manuscript and asked the additional co-author, who is a statistician, to also read the document in order to identify the unspecified persistent “grammatical errors” and “colloquialisms” and made changes where appropriate. Without explicit specification, we do not know what other errors and colloquialisms the reviewer might be referring to.*

**Comment R 1.5:** Discussion of perceived v. actual land cover (lines 299-317) – what about potential influence of PBA members due to their higher response rate (i.e. is it possible for PBA members to be more aware of woody encroachment)?

***Response R1.5:*** *See response AE 1.1. above.*

**Comment R 1.6:** Clarify or expand on “doublethink and policy-ecology mismatches” (lines 313-314)

***Response R1.6:*** *This is a quotation from a cited reference which stated: “Mismatches between policy and ecology can lead natural resource agencies toward a “doublethink” mentality (where contradictory thoughts exist without acknowledged cognitive dissonance) that produces policies that simultaneously promote and control invasive species.” We have replaced the above quote with an expanded explanation based on this statement.*

*The new wording reads “Additionally, Roberts et al. (2018) discovered that nearly all policies aimed at containing juniper tend to promote rather than contain the spread of the species due to mismatches between policy and ecology and contradictory programs within natural resources agencies that simultaneously promote and control invasive species”*

**Comment R 1.7:** Be sure to relate discussion of prescribed fire and woody plant cover back to this research. Lines 354-372 focus on other research; there is an opportunity to relate discussion about PBAs (lines 365-366) to this research (Table 7)

***Response R1.7:*** *The line numbers to which the reviewer refers are not included in the revised version of the manuscript that goes only to line 368.*

**Comment R 1.8:** Lines 375-376 – include reference

***Response R1.8:*** *Unclear to what the reviewer is referring – in our manuscript the maximum line number is 368.*

**Comment R1.9:** Conclusion paragraph (lines 381-392) – be sure to clearly summarize relationships to the three research questions and how this relates to opportunities for future research opportunities.

***Response R1.9:*** *The concluding paragraph has been revised to summarize the relationship to the three research questions and to indicate opportunities for future research.*

**Reviewer Comment 3.1:** The authors have not adequately addressed the concern raised in my review (which was also raised by Reviewer 1) that the addition of purposively sampled members of PBAs to a random sample of landowners results in a sample that, when analyzed as a single data set, is not random. To be a truly random sample, every subject in the target population must have an equal chance of being selected in the sample. In the case of the combined random + PBA member data set, this is clearly not the case. I understand the reason for purposively sampling PBA members, as there are relatively few of them, but to include them in the overall analysis violates this key assumption. This is not acknowledged in the revised manuscript and no attempt was made to correct for this in the analyses.

***Response R3.1:*** *See response AE 1.1. above.*

**Reviewer Comment 3.2:** The other possible sources of bias were evidently difficult to eliminate (mail survey – possible age bias), but the authors make a reasonable case in their rebuttal. However, this information which is of interest to the reader is not included in the revised text. All they have done is to state “Due to this limitation, we do NOT extrapolate our results to all landowners in the Southern Plains.” This may well be honest but if the aim was not to extrapolate to the bigger population, why conduct a massive survey?

***Response R3.2:*** *See response AE 1.2 above.*

**Reviewer Comment 3.3:** I understand that the data collection can no longer be changed, and that the data generated from the survey are still valuable and deserve to be published. Nevertheless, in analyzing the data, and reporting and interpreting the results, care needs to be taken to acknowledge and deal with the issues. For example, I commented: R3.28: Line 204: But this figure is influenced by the fact that PBA members were actively targeted. What is the rate of PBA membership actually like among landowners? The “figure” referred to was the value of 32 % in the sentence “About a third of respondents (32%) reported being a member of a PBA.” The percentage of PBA members relative to the general population is inflated by the inclusion of a non-random proportion of PBA members. So what then is the use of knowing that 32 % of the sample are self-reported PBA members? It does not reflect the true percentage of PBA members among the general population. Can this value be used to interpret findings that PBA membership significantly influences attitudes to fire management? I think the authors need to make a better effort (perhaps with the support of a statistician or data analyst) to address this concern, so that the way the data are reported and interpreted is robust and can allow the reader to draw conclusions about land owners in the greater region, with the appropriate caveats if required. I am not a statistician and while I can see the problem, I cannot offer an alternative way of analyzing the data that gets around the assumption of a random sample – but a statistician or data analyst would no doubt be able to help.

***Response R3.3:*** *We have consulted with a statistician, who is now a co-author of the manuscript, about the potential bias concerns. The statistician helped us craft revisions in the manuscript and the preceding responses to address this issue to the extent possible. We have also addressed these issues in a limitations and future research paragraph at the end of the discussion section.*
