# Supplementary material for: Landowner perceptions of woody plants and prescribed fire in the Southern Plains, USA
Source: PLoS One. 2020 Sep 8;15(9):e0238688. doi: 10.1371/journal.pone.0238688 (PMC7478812; doi:10.1371/journal.pone.0238688)
Supplement: Attachment — Submitted filename: Reviewer response 8-20-2020.docx [file pone.0238688.s005.docx]

Response to reviewer comments

**Editor comment:**

The authors have engaged in a major revision of the study. Although some differences remain between the reviewers' identified criticisms and the authors' approach to dealing with them, I deemed these to be acceptable.

As a minor revision, I will suggest the authors have a separate 'Conclusions', from a 'Discussion and Conclusion', section. This would largely be the last paragraph in the current section.

As the authors engage in this minor revision, I will also ask them to try to offer clear answers in the Conclusions section to their three specific questions: 1) Do landowner perceptions about woody plant expansion correspond with estimated changes in their area? 2) Do landowners within the Southern Plains desire less woody cover on their land? 3) Do expressed woody cover preferences influence the adoption of prescribed fire on private land?

This might seem like a small change but it will help with readability and the potential impact of this paper.

**Response:** We have made the requested changes in the manuscript, focusing the conclusion section on the 3 research questions.
